# Financial Toxicity and Its Association With Health-Related Quality of Life Among Partners of Colorectal Cancer Survivors

**DOI:** 10.1001/jamanetworkopen.2023.5897

**Published:** 2023-04-06

**Authors:** Lauren V. Ghazal, Paul Abrahamse, Kevin C. Ward, Arden M. Morris, Sarah T. Hawley, Christine M. Veenstra

**Affiliations:** 1 Department of Systems, Populations, and Leadership, School of Nursing, University of Michigan, Ann Arbor; 2 Department of Biostatistics, University of Michigan, Ann Arbor; 3 Department of Epidemiology, Rollins School of Public Health, Emory University, Atlanta, Georgia; 4 Department of Surgery, Stanford University, Stanford, California; 5 Division of General Medicine, Department of Internal Medicine, University of Michigan, Ann Arbor; 6 Division of Hematology/Oncology, Department of Internal Medicine, University of Michigan, Ann Arbor

## Abstract

**Importance:**

Partners of colorectal cancer (CRC) survivors play a critical role in diagnosis, treatment, and survivorship. While financial toxicity (FT) is well documented among patients with CRC, little is known about long-term FT and its association with health-related quality of life (HRQoL) among their partners.

**Objective:**

To understand long-term FT and its association with HRQoL among partners of CRC survivors.

**Design, Setting, and Participants:**

This survey study incorporating a mixed-methods design consisted of a mailed dyadic survey with closed- and open-ended responses. In 2019 and 2020, we surveyed survivors who were 1 to 5 years from a stage III CRC diagnosis and included a separate survey for their partners. Patients were recruited from a rural community oncology practice in Montana, an academic cancer center in Michigan, and the Georgia Cancer Registry. Data analysis was performed from February 2022 to January 2023.

**Exposures:**

Three components of FT, including financial burden, debt, and financial worry.

**Main Outcomes and Measures:**

Financial burden was assessed with the Personal Financial Burden scale, whereas debt and financial worry were each assessed with a single survey item. We measured HRQoL using the PROMIS-29+2 Profile, version 2.1. We used multivariable regression analysis to assess associations of FT with individual domains of HRQoL. We used thematic analysis to explore partner perspectives on FT, and we merged quantitative and qualitative findings to explain the association between FT and HRQoL.

**Results:**

Of the 986 patients eligible for this study, 501 (50.8%) returned surveys. A total of 428 patients (85.4%) reported having a partner, and 311 partners (72.6%) returned surveys. Four partner surveys were returned without a corresponding patient survey, resulting in a total of 307 patient-partner dyads for this analysis. Among the 307 partners, 166 (56.1%) were aged younger than 65 years (mean [SD] age, 63.7 [11.1] years), 189 (62.6%) were women, and 263 (85.7%) were White. Most partners (209 [68.1%]) reported adverse financial outcomes. High financial burden was associated with worse HRQoL in the pain interference domain (mean [SE] score, −0.08 [0.04]; *P* = .03). Debt was associated with worse HRQoL in the sleep disturbance domain (−0.32 [0.15]; *P* = .03). High financial worry was associated with worse HRQoL in the social functioning (mean [SE] score, −0.37 [0.13]; *P* = .005), fatigue (−0.33 [0.15]; *P* = .03), and pain interference (−0.33 [0.14]; *P* = .02) domains. Qualitative findings revealed that in addition to systems-level factors, individual-level behavioral factors were associated with partner financial outcomes and HRQoL.

**Conclusions and Relevance:**

This survey study found that partners of CRC survivors experienced long-term FT that was associated with worse HRQoL. Multilevel interventions for both patients and partners are needed to address factors at individual and systemic levels and incorporate behavioral approaches.

## Key Points

**Question:**

What is the association between financial toxicity (FT) and health-related quality of life (HRQoL) among partners of colorectal cancer survivors?

**Findings:**

In this survey study with 307 patient-partner dyads, partners with financial burden, debt, and financial worry reported poorer HRQoL in the domains of pain interference, fatigue, physical function, sleep disturbance, and social functioning. Systems- and individual-level behavioral factors were associated with partner financial outcomes and HRQoL.

**Meaning:**

These findings suggest that long-term FT is associated with poor HRQoL among partners and that interventions are critically needed for both patients and partners to address FT at multiple levels.

## Teaser

This survey study evaluates the association between financial toxicity and health-related quality of life among colorectal cancer survivors and their partners.

## Introduction

Spouses and intimate partners of cancer survivors play a critical role in diagnosis, treatment, and survivorship. While partner involvement in providing emotional, informational, and tangible support throughout the cancer trajectory is associated with clinically meaningful outcomes,^1,2,3^ the impact of cancer on partners is understudied. Financial toxicity (FT)—the protracted and sometimes overwhelming negative consequence of the cost of cancer and its treatment—is well documented among cancer survivors^4,5,6^ and is associated with poorer health-related quality of life (HRQoL).^7^ Due to material, psychosocial, and physical hardships experienced by cancer survivors,^8^ cancer-related FT may extend to their partners as well and persist long term (ie, months to years after diagnosis). However, little is known about partner FT and its possible association with partner HRQoL.

Understanding the association between FT and HRQoL among partners is critical to developing interventions to mitigate detrimental outcomes resulting from FT. While studies investigating the association between wealth and health at the household level imply an impact on a partner living in the same household,^9,10^ data collected from partners themselves are necessary to fully understand partner FT-related outcomes. Colorectal cancer (CRC) is an ideal condition in which to study this association because it is the third-most common cancer, with more than 150 000 new diagnoses annually^11^; it is diagnosed among men and women equally^12^; and its incidence among younger individuals is increasing.^13,14^ Curative-intent treatment for stage III CRC typically requires both major surgery and 3 to 6 months of chemotherapy. The physical, psychosocial, and time demands of treatment may disrupt the work and daily life of patients. Partners of patients with CRC engage in their cancer care and survivorship in myriad ways^15^ and may be similarly affected, yet FT among partners is a research area that is essentially absent in CRC populations. Thus, the purpose of this study was to assess long-term (1-5 years from diagnosis) FT among partners of CRC survivors and to understand the association between FT and partner HRQoL.

## Methods

### Conceptual Framework

The expanded^16^ FT framework of the National Cancer Institute^4^ guided our analysis, and we further adapted it to include partners (eFigure in Supplement 1).

### Study Design, Setting, and Participants

Using a convergent mixed-methods study design and following the American Association for Public Opinion Research (AAPOR) reporting standards, we simultaneously collected quantitative and qualitative data through a survey of patients and partners, analyzed responses to closed- and open-ended items independently, and then merged the findings.^17,18,19^ We collected qualitative data to further confirm and identify mechanistic factors leading to outcomes in the quantitative analysis.^20,21^

Surveys were distributed in multiple waves from April 2019 to February 2020, using a modified Dillman approach.^22^ We identified patients aged 21 to 85 years who underwent surgical resection of stage III CRC in 2014 to 2018 via the tumor registries of a community oncology practice (Billings Clinic, Billings, Montana), an academic cancer center (Rogel Cancer Center, Ann Arbor, Michigan), and archival data from the Georgia Cancer Registry for patients across the state. Patients were surveyed 1 to 5 years after cancer diagnosis. Patients with known cancer recurrence or progression were ineligible. Patient-identified partners (spouse, domestic partner, or significant other) living in the same household were eligible for this study.

Patients were mailed a patient survey packet with a $10 cash gift and a separate partner survey packet to give to their partner. Upon receipt of their completed survey, partners were mailed a $10 cash gift. Completed surveys from patients and their partners were linked using unique identification numbers. The institutional review boards at all study sites approved this study and waived the need for written informed consent. Return of a completed survey was considered to be implied consent to participate. Patients and partners who did not wish to participate were free to choose not to complete or return a survey.

### Measures

Measures were developed based on our conceptual framework^4,16^ and the existing literature.^23,24,25^ We used standard techniques to assess content validity, including expert reviews as well as cognitive pretesting and pilot testing of measures among CRC survivors and partners.^17^

#### Quantitative Outcomes

##### FT Outcomes

Based on our conceptual framework, we assessed 3 intermediate FT outcomes among partners: financial burden, debt, and financial worry.

We measured financial burden using the Personal Financial Burden scale previously developed by our team.^23^ This measure includes multiple items (with yes or no responses) that assess financial burdens, such as cutting down on spending and missing credit card or bill payments, “due to the financial impact of (the patient) having colorectal cancer.” Responses were scored on a continuous scale, with a higher score indicating higher burden.

Debt was measured with a single item on the survey (yes or no): “Do you currently have debt (for example, unpaid bills, credit card balance, bank loans, or borrowing money from family or friends) from (patient’s) colorectal cancer treatment?”

Financial worry was measured with a single item on the survey: “How much do you worry about current or future financial problems as a result of (patient’s) colorectal cancer and treatments?” Consistent with prior work, we collected responses using a 5-point Likert scale (with 1 indicating not at all and 5 indicating a lot) and dichotomized them into high and low worry.^23,24^

##### HRQoL Outcomes

We used the PROMIS-29+2 Profile, version 2.1 (29-item Patient-Reported Outcomes Measurement Information System Profile, with 2 cognitive function abilities items),^25,26,27^ to capture a comprehensive, subjective appraisal of HRQoL across 7 domains: physical function, anxiety, depression, fatigue, sleep disturbance, social roles and activities, and pain interference and intensity. Higher scores in each domain represented better quality of life. Domain scores were obtained from HealthMeasures Scoring Services.^28^

#### Covariates

##### Sociodemographic and Clinical Factors

Patients and partners self-reported their age, sex, race and ethnicity, education level, comorbid conditions, and employment status at the time of patient diagnosis. All participants self-reported their race and ethnicity as Black, White, or other (American Indian or Alaska Native, Asian Indian, Chinese, Filipino, Japanese, Korean, Native Hawaiian or other Pacific Islander, or Vietnamese). Because of expected multicollinearity between partner and patient sociodemographic factors, only patient-reported annual household income and relevant clinical factors (primary cancer site, receipt of chemotherapy, and receipt of radiation) were included in these analyses.^29^

##### Financial Cost and Partner Perceptions of Financial Roles

Partners reported lost income and missed workdays due to their partners’ cancer. We hypothesized that partners’ financial role may be associated with FT, and we measured financial roles using 2 survey items: “During [patient’s] cancer care, (1) How much were you helping them financially? (2) How much were they helping you financially?” Responses were on a 5-point Likert scale (with 1 indicating not at all and 5 indicating a lot) and were dichotomized into high and low levels of helping.

#### Qualitative Data

Patient and partner surveys culminated with an open-ended question, asking respondents to share their experiences with cancer and its treatment.

#### Missing Data

There were few missing values (<3.0%) for all variables except annual household income, for which 41 patients (13.4%) did not respond or reported they did not know. Multiple imputation techniques were used to account for missing annual household income data.^30,31^

### Statistical Analysis

#### Descriptive Analysis

We performed descriptive analyses of the 3 partner-reported FT intermediate outcomes. Using analysis of variance and χ^2^ tests, we evaluated bivariate associations between each FT intermediate outcome and independent partner and patient variables. We then estimated 3 multivariable regressions to evaluate associations between partner FT intermediate outcomes and HRQoL domains, as well as partner and patient variables, adjusted for covariates. To reduce potential nonresponse bias, weights were created with a logistic regression of partner nonresponse on demographic characteristics of patients and used in multivariable analyses. All statistical tests were 2 sided; *P* < .05 was considered significant. Analyses were conducted with SAS, version 9.4 (SAS Institute). Data analysis was performed from February 2022 to January 2023.

#### Qualitative and Mixed-Methods Analysis

To ensure rigor and validity, open-ended responses were transferred verbatim from surveys into a secure REDCap (Research Electronic Data Capture) database and combined in a master document. All finance-related responses were compiled and reduced using a matrix method.^32^ We used thematic analysis to identify main responses across all free-text open responses related to the 3 FT intermediate outcomes.^33,34^

We integrated quantitative and qualitative findings to enrich understanding. To enhance credibility and establish rigor, 2 research team members (L.V.G. and C.M.V.) reviewed the data, coded them separately, and met regularly to achieve consensus on the qualitative data and their integration with the quantitative data, including disconfirming (ie, contradictory) evidence. We present results of the quantitative analysis as well as illustrative quotes and meta-inferences (ie, conclusions generated through integration of qualitative and quantitative data).^35,36^

## Results

### Patient and Partner Characteristics

Among the 986 patients with CRC who were eligible for this survey study, 501 (50.8%) returned surveys. Of these 501 patients, 266 (53.1%) were aged younger than 65 years; 215 (42.9%) were women and 286 (57.1%) were men; and 371 (74.1%) had at least some college education. A total of 53 patients (10.6%) were Black, 412 (82.2%) were White, and 36 (7.2%) were of other race and ethnicity. More than half of the patients (291 [58.1%]) were within 3 to 4 years from diagnosis. Among the 501 respondents, 428 patients (85.4%) reported having a partner, and 311 partners (72.6%) returned surveys. Among these 311 partners, 169 (54.3%) were aged younger than 65 years; 192 (61.7%) were women and 118 (37.9%) were men [1 partner who was not linked to a patient was missing a reported gender]; and 225 (72.3%) had at least some college education. A total of 20 partners (6.4%) were Black, 267 (85.9%) were White, and 24 (7.7%) were of other race and ethnicity. Of the 167 partners (53.7%) who were employed full- or part-time at the time of their partner’s CRC diagnosis, 65 (38.9%) missed 7 to 30 days of work, and 64 (38.3%) reported lost income due to the patient’s cancer.

Four partner surveys were returned without a corresponding patient survey; therefore, paired surveys from 307 patient-partner dyads were included in these analyses (Table 1). For patients and partners, the mean (SD) age was 62.7 (11.3) vs 63.7 (11.1) years. A total of 111 patients (36.3%) were women and 195 (63.7%) were men; 189 partners (62.6%) were women and 113 (37.4%) were men. Patients and partners self-reported race and ethnicity as Black (23 [7.5%] vs 20 [6.5%]), White (262 [85.3%] vs 263 [85.7]), or other (22 [7.5%] vs 24 [7.8%]).

**Table 1.** Characteristics of Patient-Partner Dyads^a^

Characteristic	Patients (n = 307)	Partners (n = 307)
Age, y		
Mean (SD)	62.7 (11.3)	63. 7 (11.1)
<50	36 (11.8)	48 (16.2)
51-64	128 (41.8)	118 (39.9)
≥65	142 (46.4)	130 (43.9)
Missing	1 (0.3)	11 (3.6)
Sex		
Female	111 (36.3)	189 (62.6)
Male	195 (63.7)	113 (37.4)
Missing	1 (0.3)	5 (0.9)
Race and ethnicity		
Black	23 (7.5)	20 (6.5)
White	262 (85.3)	263 (85.7)
Other^b^	22 (7.5)	24 (7.8)
Educational attainment		
High school or less	70 (22.8)	82 (26.7)
Some college	104 (33.9)	108 (35.2)
College graduate	133 (43.3)	114 (37.1)
Missing	0	3 (1.0)
Comorbid conditions		
0	87 (28.3)	78 (25.4)
≥1	220 (71.7)	229 (74.6)
Employment status at time of patient diagnosis		
Full-time	151 (49.2)	128 (41.7)
Part-time	10 (3.3)	35 (11.4)
Retired	109 (35.5)	100 (32.6)
Other^c^	37 (12.1)	44 (14.3)
Missing	0	0
Annual household income, $^d^		
<40 000	94 (35.3)	NA
40 000-89 999	56 (21.1)	NA
≥90 000	116 (43.6)	NA
Missing	41 (13.4)	NA
Lost income among partners, $^e^		
0	NA	82 (53.6)
1-5000	NA	41 (26.8)
≥5001	NA	30 (19.6)
Missing	NA	21 (6.8)
Lost work among partners^e^		
≤1 week	NA	60 (38.7)
7-30 d	NA	68 (43.9)
>1 mo	NA	27 (17.4)
Missing	NA	19 (6.2)^a^
Patient receipt of chemotherapy		
No	16 (5.3)	NA
Yes	285 (94.7)	NA
Missing	6 (2.0)	NA
Patient receipt of radiation therapy		
No	198 (66.0)	NA
Yes	102 (34.0)	NA
Missing	7 (2.3)	NA
Patient-reported cancer site		
Colon	187 (60.9)	NA
Rectum	40 (13.0)	NA
Both or unknown	80 (26.1)	NA
Years since patient diagnosis		
1-2	78 (26.4)	NA
3-4	182 (61.5)	NA
≥5	36 (12.2)	NA
Missing	11 (3.6)	NA

Abbreviation: NA, not applicable.

^a^ Unless indicated otherwise, data are reported as No. (%) of patients or partners. The participant sample consisted of 307 patient-partner dyads from 3 settings: tumor registries of a community oncology practice (n = 4), an academic cancer center (n = 86), and archival registry data from the Georgia Cancer Registry (n = 217).

^b^ Includes American Indian or Alaska Native, Asian Indian, Chinese, Filipino, Japanese, Korean, Native Hawaiian or other Pacific Islander, and Vietnamese.

^c^ Includes students or individuals who had a disability, were unemployed and looking for work, or were temporarily laid off or on sick or other leave.

^d^ As reported by the patient.

^e^ Exclusive of the 134 partners who were not working at the time of patient’s diagnosis.

We observed that patient response rates were significantly lower for Black and other racial and ethnic minority individuals (χ^2^ = 4.5, *P* = .04), men (χ^2^ = 4.1, *P* = .04), and Georgia Cancer Registry–identified patients (χ^2^ = 18.3, *P* < .001). Patients without a partner were more likely to be women (χ^2^ = 10.5, *P* = .001) or Black (χ^2^ = 16.3, *P* = .003).

### Partner FT Intermediate Outcomes

#### Financial Burden

The mean (SD) financial burden score for partners was 2.06 (1.90). Among the 307 partners, 193 (62.9%) experienced 1 or more of the financial burden items; 156 (50.8%) reported that they cut down on expenses, 143 (46.6%) cut down on recreational activities, 116 (37.8%) used savings, and 119 (38.8%) cut down on spending for food or clothes (eTable 1 in Supplement 1). In bivariate analyses (Table 2), partners who were younger (*F* = 9.4, *P* < .001), employed (*F* = 5.24, *P* = .002), had greater lost income (*F* = 19.6, *P* < .001), had greater missed work (*F* = 10.1, *P* < .001), and had high levels of helping the patient financially (*F* = 9.1, *P* < .001) were more likely to report higher financial burden.

**Table 2.** Bivariate Analyses of Financial Burden Among Partners^a^

Characteristic	Financial burden score (n = 307)^b^	*P* value
Age, y		
<50	2.55 (1.80)	<.001
51-64	2.47 (1.81)	
≥65	1.47 (2.07)	
Sex		
Female	2.38 (1.94)	.73
Male	2.33 (1.74)	
Race and ethnicity		
Black	2.08 (2.95)	.02
White	2.35 (1.82)	
Other^c^	2.14 (1.86)	
Education		
High school or less	2.61 (1.97)	.96
Some college	2.21 (1.66)	
College graduate	2.38 (1.97)	
Comorbid conditions		
0	2.30 (1.68)	.81
≥1	2.38 (1.95)	
Employment status at time of patient diagnosis		
Full-time	2.37 (1.86)	.002
Part-time	2.17 (1.76)	
Retired	2.75 (2.22)	
Other^d^	2.50 (2.65)	
Annual household income, $^e^		
<40 000	3.21 (1.98)	.02
40 000-89 999	2.25 (1.78)	
≥90 000	2.12 (1.80)	
Lost income, $		
0	1.78 (1.68)	<.001
1-5000	2.40 (1.90)	
≥5001	3.92 (1.38)	
Lost work		
≤1 week	1.81 (1.76)	<.001
7-30 d	2.33 (1.78)	
>1 mo	3.67 (1.71)	
Partner helping patient financially		
High	2.40 (1.87)	<.001
Low	2.31 (1.87)	
Patient helping partner financially		
High	2.14 (1.73)	.12
Low	2.59 (1.98)	
Patient receipt of chemotherapy		
No	2.67 (0.82)	.21
Yes	2.34 (1.90)	
Patient receipt of radiation therapy		
No	2.18 (1.82)	.19
Yes	2.66 (1.91)	
Patient-reported cancer site		
Colon	2.21 (1.85)	.28
Rectum	2.35 (1.80)	
Both or unknown	2.71 (1.94)	
Years since patient diagnosis		
1-2	2.03 (1.84)	.36
3-4	2.73 (1.80)	
≥5	1.42 (1.80)	

^a^ For the ordinal covariates, we also tested for trend and did not find any meaningful differences in significance.

^b^ A higher mean (SD) score corresponds to a greater (worse) financial burden.

^c^ Includes American Indian or Alaska Native, Asian Indian, Chinese, Filipino, Japanese, Korean, Native Hawaiian or other Pacific Islander, or Vietnamese.

^d^ Includes students or individuals who had a disability, were unemployed and looking for work, or were temporarily laid off or on sick or other leave.

^e^ As reported by the patient.

#### Debt

A total of 88 partners (28.6%) reported current cancer-related debt. Partners with lower patient-reported annual household income (χ^2^ = 8.0, *P* = .02), 1 or more comorbid conditions (χ^2^ = 7.9, *P* = .005), greater lost income (χ^2^ = 19.3, *P* < .001), more missed work (χ^2^ = 9.0, *P* = .01), and high levels of helping the patient financially (χ^2^ = 7.4, *P* = .006) were significantly more likely to report debt (Table 3).

**Table 3.** Bivariate Analyses of Debt and Financial Worry Among Partners^a^

Characteristic	Current debt			Financial worry		
	Yes (n = 88)	No (n = 211)	*P* value	High (n = 109)	Low (n = 189)	*P* value
Age, y						
<50	20 (23.2)	28 (13.7)	.02	22 (21.0)	26 (14.1)	.09
51-64	38 (44.2)	76 (37.3)		46 (43.8)	71 (38.4)	
≥65	28 (32.6)	100 (49)		37 (35.2)	88 (47.6)	
Sex						
Female	51 (57.3)	137 (65.6)	.31	69 (64.5)	118 (62.4)	.73
Male	35 (40.7)	72 (34.4)		38 (35.5)	71 (37.6)	
Race and ethnicity						
Black	9 (10.2)	11 (5.2)	.19	5 (4.6)	14 (7.4)	.09
White	71 (80.7)	186 (88.2)		92 (84.4)	166 (87.8)	
Other^b^	8 (9.1)	14 (6.6)		12 (11.0)	9 (4.8)	
Education						
High school or less	29 (32.9)	51 (24.4)	.20	30 (27.5)	49 (25.9)	.80
Some college	32 (36.4)	74 (35.4)		40 (36.7)	65 (34.4)	
College graduate	27 (30.7)	84 (40.2)		39 (35.8)	75 (39.7)	
Comorbid conditions						
0	12 (13.6)	61 (28.9)	.005	23 (21.1)	52 (27.5)	.22
≥1	76 (86.4)	150 (71.1)		86 (78.9)	137 (72.5)	
Employment status at time of patient diagnosis						
Full-time	43 (48.9)	82 (38.9)	.09	54 (49.5)	72 (38.1)	.04
Part-time	8 (9.1)	27 (12.8)		10 (9.2)	24 (12.7)	
Retired	21 (23.9)	76 (36)		26 (23.9)	70 (37)	
Other^c^	16 (18.1)	26 (12.3)		19 (17.4)	23 (12.2)	
Annual household income, $						
<40 000	32 (40.0)	60 (33.1)	.02	37 (37.0)	55 (33.5)	.27
40 000-89 999	23 (28.8)	32 (17.7)		25 (25.0)	31 (18.9)	
≥90 000	25 (31.2)	89 (49.2)		38 (38.0)	78 (47.6)	
Lost income, $						
0	17 (34.7)	64 (63.4)	<.001	21 (34.4)	59 (65.6)	<.001
1-5000	13 (26.5)	27 (26.7)		19 (31.2)	22 (24.4)	
≥5001	19 (38.8)	10 (9.9)		21 (34.4)	9 (10)	
Lost work						
≤1 week	11 (23.4)	48 (45.7)	.01	13 (21.3)	44 (48.4)	<.001
7-30 d	23 (48.9)	44 (41.9)		30 (49.2)	38 (41.8)	
>1 mo	13 (27.7)	13 (12.4)		18 (29.5)	9 (9.9)	
Partner helping patient financially						
High	55 (62.5)	92 (45.1)	.006	61 (56.0)	86 (47.3)	.15
Low	33 (37.5)	112 (54.9)		48 (44.0)	96 (52.7)	
Patient helping partner financially						
High	52 (59.1)	103 (50.5)	.18	57 (52.3)	96 (52.7)	.94
Low	36 (40.9)	101 (49.5)		52 (47.7)	86 (47.3)	
Patient receipt of chemotherapy						
No	4 (4.7)	103 (25.1)	.95	5 (4.8)	96 (26)	.98
Yes	82 (95.3)	101 (24.6)		100 (95.2)	86 (23.3)	
Patient receipt of radiation therapy						
No	54 (64.3)	139 (66.8)	.68	66 (62.9)	124 (66.7)	.51
Yes	30 (35.7)	69 (33.2)		39 (37.1)	62 (33.3)	
Patient-reported cancer site						
Colon	53 (60.2)	130 (61.6)	.96	64 (58.7)	116 (61.4)	.90
Rectum	11 (12.5)	27 (12.8)		15 (13.8)	24 (12.7)	
Both or unknown	24 (27.3)	54 (25.6)		30 (27.5)	49 (25.9)	
Years since patient diagnosis						
1-2	21 (25.3)	56 (27.1)	.08	24 (22.9)	52 (28.4)	.05
3-4	57 (68.7)	120 (58)		73 (69.5)	103 (56.3)	
≥5	5 (6.0)	31 (15)		8 (7.6)	28 (15.3)	

^a^ Unless indicated otherwise, data are reported as No. (%) of partners. For the ordinal covariates, we also tested for trend and did not find any meaningful differences in significance.

^b^ Includes American Indian or Alaska Native, Asian Indian, Chinese, Filipino, Japanese, Korean, Native Hawaiian or other Pacific Islander, or Vietnamese.

^c^ Includes individuals who had a disability, were unemployed and looking for work, or were temporarily laid off or on sick or other leave.

#### Financial Worry

A total of 109 partners (35.5%) reported high financial worry. Partners who were employed (χ^2^ = 7.9, *P* = .04), had greater lost income (χ^2^ = 37.5, *P* < .001), and had more missed work (χ^2^ = 24.0, *P* < .001) were significantly more likely to report high financial worry (Table 3).

Multivariable analyses of the association of FT intermediate outcomes with partner and patient variables suggested similar findings (eTable 2 in Supplement 1).

### Partner HRQoL

Results of multivariable analyses of the association between each of the 3 FT intermediate outcomes and partner HRQoL are displayed in the Figure and in eTable 3 in Supplement 1. Separate generalized linear models were created for each partner HRQoL dimension, using the 3 FT intermediate outcomes as predictors and including the partner demographic variables and patient clinical variables listed in Table 1 as covariates. Across the 7 HRQoL domains, greater financial burden was associated with worse pain interference (mean [SE] score, −0.08 [0.04]; *P* = .03). Debt was associated with worse sleep disturbance (mean [SE] score, −0.32 [0.15]; *P* = .03). High financial worry was associated with worse social functioning (mean [SE] score, −0.37 [0.13]; *P* = .005), fatigue (−0.33 [0.15]; *P* = .03), and pain interference (−0.33 [0.14]; *P* = .02). For each of these, the difference between low and high financial impact was associated with a 0.4 SD or greater decrease in HRQoL.

**Figure.**
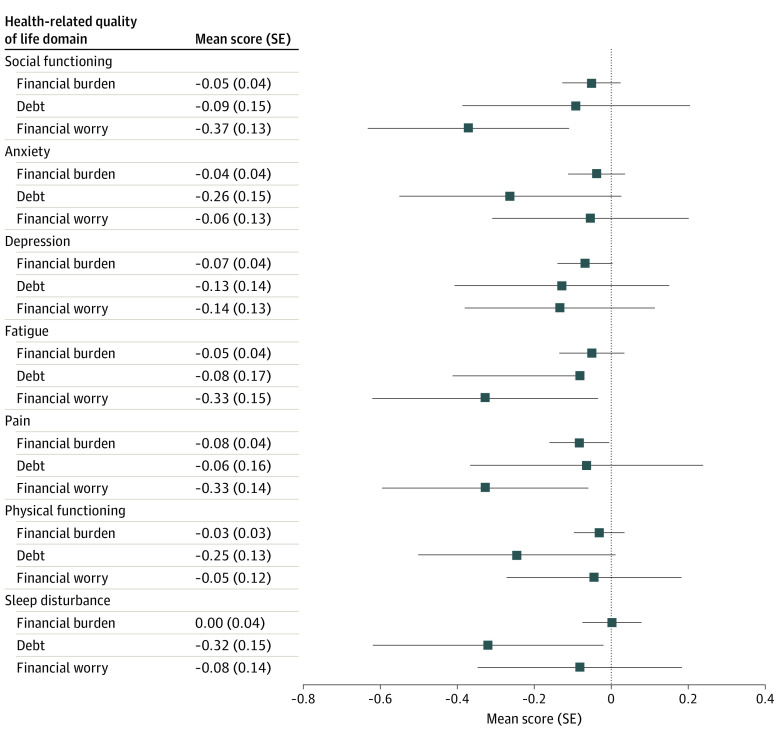
Associations Between Health-Related Quality of Life Domains and Financial Toxicity Outcomes

### Qualitative and Mixed-Methods Results

Partners described systems-level factors contributing to FT, such as high costs, multiple bills from different providers, challenges with health insurance, missed or lost work and wages, and the “daunting” insurance and health care systems. Most partners described FT of cancer as dependent on health insurance and employment status. Partners also described individual-level factors contributing to FT, such as uncharacteristic spending to cope with the cancer-related emotional burden, questioning what might have happened without insurance or savings, and disruption to social relationships as they asked for material support for medications, medical supplies, and living costs. Partners described systems-level issues as primary drivers of FT, which led to individual, behavioral mechanisms around financial decision-making and coping.

The quantitative (Figure) and qualitative findings were integrated to derive meta-inferences that provided insight into the quantitative findings (Table 4). The qualitative findings suggested that the impact of financial burden on HRQoL was substantial. For example, one partner stated that the cancer diagnosis caused them to use savings (“sell off retirement dreams”), sell belongings for money, and participate in financial crowdsourcing for medicines, medical supplies, and housing. Partners described debt-related mental health outcomes such as depression and “[spending] a lot of money to cope” with cancer. In addition, partners described the isolating nature of financial worry. For example, one partner “often felt alone and left to figure things out without a support system” after the patient lost their job due to cancer. Another reported “negative effects to intimacy” stemming from worry and finances. Many partners described gratitude surrounding conditional situations that afforded them protection against financial hardship. However, some reported worrying about the FT they might have faced without adequate employment, health insurance, or savings.

**Table 4.** Qualitative Themes and Meta-inferences Regarding Financial Toxicity Outcomes and Health-Related Quality of Life Among Partners of Colorectal Cancer Survivors

Financial toxicity outcome	Illustrative quote	Meta-inference
Financial burden	“We had to sell off our retirement dreams. We had to sell anything/everything of value to survive, then had to ask family members and friends for help to stay in our home, get medicines prescribed, to purchase test preps even!”	Financial burden was associated with worse HRQoL in pain interference. Qualitative findings indicate that for partners who used extreme mechanisms of financial coping, the changes in HRQoL were substantial. Material resources were exhausted, and social relationships were strained by requests for financial assistance.
Debt	“[Patient] was depressed during that year and spent a lot of money to cope which is why we have debt.”	Debt was significantly associated with worse HRQoL in sleep disturbance. In addition to costs of care, partners may have spent money as a coping mechanism, leading to debt.
	“Setting up payment plans with all the providers was overwhelming since most didn’t accept … hassles for payments on the phone made me never want to answer the phone.”	
Financial worry	“The overall impact of multiple bills from every provider … was never ending.”	Financial worry was associated with worse HRQoL in social, fatigue, and pain interference. For some partners, the worry was isolating and made them feel “alone … without a support system.” Even partners with adequate finances expressed gratitude surrounding their conditional situations that afforded them protection and often worried about the financial toxicity they would have incurred without these protections.
	“The cancer diagnosis wrecked our lives. It felt like everything was happening in a whirlwind while also painstakingly slow.”	
	“Health care cost and financials … is daunting.”	
	“We were very fortunate to have access to good health insurance and [an Employee Assistance Program] which paid everything insurance didn’t cover.”	

Abbreviation: HRQoL, health-related quality of life.

## Discussion

The results of this mixed-methods survey study suggest that long-term FT is associated with decreased HRQoL among partners of CRC survivors. Using data reported by partners themselves that were obtained years after the initial patient diagnosis, we observed that more than half of partners (62.9%) experienced financial burden, nearly a third (28.6%) experienced debt, and more than a third (35.5%) experienced high financial worry. Younger partners were significantly more likely to report financial burden and debt, a finding that aligns with our prior work showing increased financial hardship among younger patients with CRC.^23,31^ In addition, this result is particularly salient given recent alarming increases in CRC incidence among patients aged younger than 50 years.^13,14^ In this study, we leveraged quantitative and qualitative data to gain insight into associations between partner FT and individual domains of HRQoL, which is an important contribution to the FT literature. The 3 FT intermediate outcomes we measured were each associated with an adverse impact on different HRQoL domains—including pain, fatigue, sleep disturbance, and social functioning—thus uncovering how individual components of FT were associated with HRQoL.

To our knowledge, this study is the first to examine partners of CRC survivors in this way and builds on prior work to demonstrate an association of long-term FT with poor HRQoL among partners. A prior single-center study of a convenience sample of adult patients with solid tumors and their caregivers reported associations between increased caregiver FT and decreased HRQoL.^37^ Another study of partners of patients with cancer in South Korea reported an association of caregivers’ lost savings and income with a negative impact on HRQoL.^38^ In a recent study of caregivers of adolescent and young adult cancer survivors, researchers observed high financial burden for 25% of caregivers, often secondary to employment disruptions and costs of care.^39^ In addition, a 2021 study of survivors of multiple cancer types reported associations between pain and negative survivor financial and employment outcomes.^40^ Our results extend beyond those of prior studies to further confirm and identify mechanistic factors in the association between FT and specific domains of partner HRQoL.

Meta-inferences help explain the quantitative associations we observed between each of the 3 FT intermediate outcomes and individual domains of HRQoL. In addition to systems-level factors such as high costs and problems navigating the health care system, partners described an additional layer of individual-level, compensatory behavioral mechanisms contributing to the outcomes across HRQoL domains. We observed that financial burden was associated with the pain domain of HRQoL. Similar to prior studies in the general adult population that show financial hardship to be the largest risk factor for debilitating pain,^41,42^ partners in our study described difficulty paying bills and obtaining medical supplies and care that caused them to turn to family and friends for financial assistance, straining these important social relationships. These factors—inability to afford medical necessities and the psychological impact of financial crowdsourcing—help explain the association we observed between financial burden and pain.

We observed that debt was associated with the sleep disturbance domain of HRQoL. The debt-related mental health outcomes that partners described—such as uncharacteristic spending to cope with the emotional burden of cancer and angst from “never-ending bills” and calls from bill collectors—help explain these associations. We also noted that financial worry was associated with the social functioning, fatigue, and pain domains of HRQoL. The isolation that partners felt due to financial worry, including loss of support systems and intimacy, was associated with negative impacts to social functioning. Worry about both actual and potential FT was associated with poor HRQoL among partners in our study. The phenomenon of worrying about FT that they could have faced is similar to, although not as extreme as, catastrophizing—the psychological process of imagining the worst possible outcome—which has been associated with fatigue and pain among individuals with chronic disease, including cancer.^43^ These important findings contribute to the FT literature, and they suggest that future interventions to mitigate FT should target the interplay of systems- and individual-level factors among both patients and partners and should incorporate more behavioral approaches to address social disruptions, uncharacteristic spending, and worry about “what could have been.”

### Limitations

This study has some limitations. We acknowledge that our findings may not apply to all partners of CRC survivors; however, we obtained broad representation from 3 diverse settings to optimize generalizability. While nonresponse bias was possible, response rates among patients (50.8%) and partners (72.6%) were comparable to or exceeded those of other large survey studies of CRC survivors and partners.^44,45^ There was a greater proportion of male patients (63.7%) than female patients (36.3%) in our analytic sample of paired patient-partner dyads. Consistent with prior work,^46^ female patients in our entire patient sample were significantly less likely to report having a partner and may be more likely to receive support from nonpartner family members or friends. Future studies should include these important nonpartner supporters. There may be unobservable variables not accounted for in our analyses. To mitigate this, we collected data on key variables in our conceptual framework, which are likely to be correlated with most unobserved variables reasonably expected to be associated with FT and HRQoL.

Although our study was observational in nature, a convergent mixed-methods design contributed to its rigor and allowed us to derive further insights into the quantitative findings. It is worth noting that in an optional, open-ended question that we purposely left broad, many partners felt compelled to handwrite responses about the impact of CRC on their finances and lives; finances were the most frequently written-about issue in these responses. Because the responses were mailed back, we were unable to “dive deeper” into them. Still, these rich qualitative data were instrumental to better understanding long-term FT among partners and associations with HRQoL.

## Conclusions

The results of this survey study suggest that long-term FT among partners of CRC survivors was associated with worse HRQoL via systems- and individual-level behavioral factors. Our findings provide an understanding of the association between FT and specific domains of partner HRQoL. This contribution to the FT literature will help researchers better tailor interventions to effectively mitigate FT and improve HRQoL for both patients and partners.

## Notes

Supplement 1. **eFigure.** Adapted Model of Financial Toxicity Among Colorectal Cancer Survivors and Their Partners **eTable 1.** Prevalence of Financial Coping Mechanisms (Financial Burden) Among Partners **eTable 2.** Multivariable Analyses of Associations Between Financial Outcomes and Partner or Patient Variables **eTable 3.** Multivariable Analyses of Associations Between Health-Related Quality of Life Domains and Financial Outcomes, Among Partners

Supplement 2.
Data Sharing Statement
