# Supplementary material for: Financial Toxicity and Its Association With Health-Related Quality of Life Among Partners of Colorectal Cancer Survivors
Source: JAMA Netw Open. 2023 Apr 6;6(4):e235897. doi: 10.1001/jamanetworkopen.2023.5897 (PMC10080378; doi:10.1001/jamanetworkopen.2023.5897)
Supplement: Supplement 1. — eFigure. Adapted Model of Financial Toxicity Among Colorectal Cancer Survivors and Their Partners eTable 1. Prevalence of Financial Coping Mechanisms (Financial Burden) Among Partners eTable 2. Multivariable Analyses of Associations Between Financial Outcomes and Partner or Patient Variables eTable 3. Multivariable Analyses of Associations Between Health-Related Quality of Life Domains and Financial Outcomes, Among Partners [file jamanetwopen-e235897-s001.pdf]

Supplementary Online Content

Ghazal LV, Abrahamse P, Ward KC, Morris AM, Hawley ST, Veenstra CM. Financial toxicity and its association with health-related quality of life among partners of colorectal cancer survivors. *JAMA Netw Open*. 2023;6(4):e235897. doi:10.1001/jamanetworkopen.2023.5897

eFigure. Adapted Model of Financial Toxicity Among Colorectal Cancer Survivors and Their Partners

eTable 1. Prevalence of Financial Coping Mechanisms (Financial Burden) Among Partners

eTable 2. Multivariable Analyses of Associations Between Financial Outcomes and Partner or Patient Variables

eTable 3. Multivariable Analyses of Associations Between Health-Related Quality of Life Domains and Financial Outcomes, Among Partners

This supplementary material has been provided by the authors to give readers additional information about their work.

© 2023 Ghazal LV et al. *JAMA Network Open*.

eFigure. Adapted Model of Financial Toxicity Among Colorectal Cancer Survivors and Their Partners

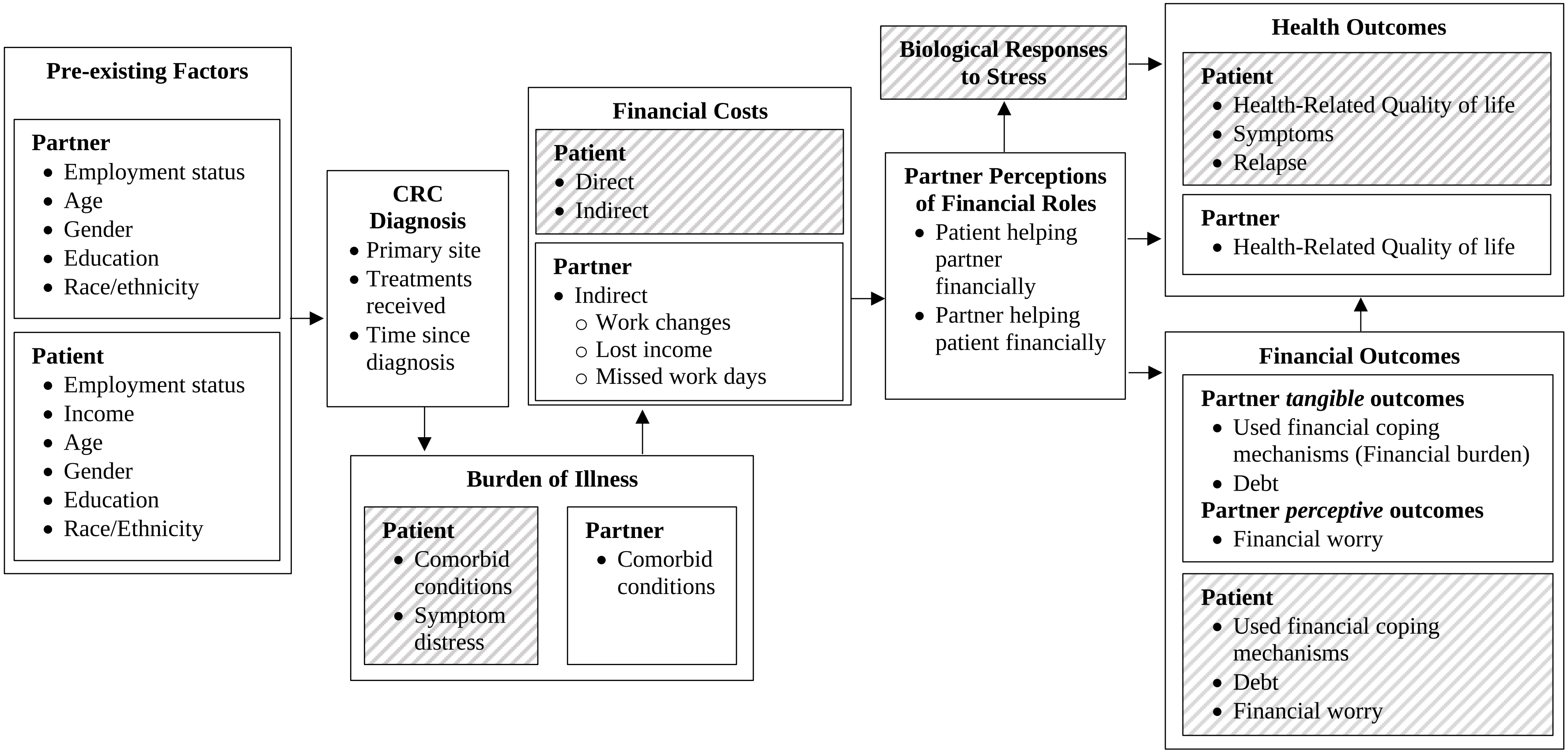

Note. Santacroce and Kneipp expanded the Ramsey and the National Cancer Institute (NCI) model to fully capture individual and ecological factors affecting risk for financial toxicity among parents who experience the diagnosis of pediatric cancer. Our adapted model illustrates pre-illness patient and partner factors that impact financial and employment outcomes, and describes relationships between pre-existing factors, financial worry, and health outcomes for adult cancer survivors and their partners. CRC= colorectal cancer. Grayed-out boxes indicate patient variables that are not analyzed in this current study, but inform the larger framework.

© 2023 Ghazal LV et al. *JAMA Network Open*.

eTable 1. Prevalence of Financial Coping Mechanisms (Financial Burden) Among Partners

Survey Item	No. (%)
I had to use savings	116 (37.8)
I could not make payments on credit cards or other bills	37 (12.1)
I cut down on spending for food and/or clothes	119 (38.8)
I had my utilities turned off because the bill was not paid	5 (1.6)
I had to move out of my house or apartment because I could not afford to stay there	3 (1.0)
I cut down on spending for health care for other family members	21 (6.8)
I cut down on recreational activities	143 (46.6)
I cut down on expenses in general	156 (50.8)

© 2023 Ghazal LV et al. *JAMA Network Open*.

eTable 2. Multivariable Analyses of Associations Between Financial Outcomes and Partner or Patient Variables

	**Financial Burden**			**Debt**			**Financial Worry**		
	Coef	SE	p	Coef	SE	p	Coef	SE	p
**Annual Household Income**									
<$40,000 (Ref)									
$40,000-$89,999	-0.336	0.130	0.010	0.184	0.239	0.442	0.148	0.217	0.496
>$90,000	-0.695	0.138	<.0001	-0.958	0.278	0.001	-0.347	0.234	0.139
**Comorbid Conditions**									
0 (Ref)									
1 Or More	0.073	0.112	0.518	0.729	0.236	0.002	0.177	0.183	0.333
**Patient Receipt of Chemotherapy**									
No (Ref)									
Yes	-0.231	0.210	0.270	0.119	0.454	0.793	-0.171	0.380	0.652
**Patient Receipt of Radiation Therapy**									
No (Ref)									
Yes	0.099	0.144	0.494	0.038	0.265	0.887	0.095	0.231	0.681
**Patient-Reported Cancer Site**									
Rectal (Ref)									
Colon	0.285	0.189	0.131	0.157	0.348	0.653	0.143	0.303	0.637
Both/Unknown	0.323	0.163	0.047	-0.123	0.297	0.679	0.105	0.254	0.680

Note. Financial burden is modeled with a Poisson model and debt and worry are modeled with logistic models. Models also controlled for partner age, gender, race, education, employment, lost income, and lost work; partner helped by patient financially, patient helped by partner financially; and years since patient diagnosis.

© 2023 Ghazal LV et al. *JAMA Network Open*.

eTable 3. Multivariable Analyses of Associations Between Health-Related Quality of Life Domains and Financial Outcomes, Among Partners

	Social		Anxiety		Depressive symptoms		Fatigue		Pain interference		Physical function		Sleep disturbance	
	Mean (SE)	*P* Value	Mean (SE)	*P* Value	Mean (SE)	*P* Value	Mean (SE)	*P* Value	Mean (SE)	*P* Value	Mean (SE)	*P* Value	Mean (SE)	*P* Value
Financial Burden														
	-0.05 (0.04)	0.18	-0.04 (0.04)	0.30	-0.07 (0.04)	0.06	-0.05 (0.04)	0.24	-0.08 (0.04)	**0.03**	-0.03 (0.03)	0.34	0.00 (0.04)	0.98
Debt														
No	REF		REF		REF		REF		REF		REF		REF	
Yes	-0.09 (0.15)	0.54	-0.26 (0.15)	0.07	-0.13 (0.14)	0.36	-0.08 (0.17)	0.63	-0.06 (0.15)	0.67	-0.25 (0.13)	0.06	-0.32 (0.15)	**0.03**
Financial Worry														
Low	REF		REF		REF		REF		REF		REF		REF	
High	-0.37 (0.13)	**0.005**	-0.06 (0.13)	0.67	-0.14 (0.13)	0.28	-0.33 (0.15)	**0.03**	-0.33 (0.14)	**0.02**	-0.05 (0.12)	0.69	-0.08 (0.14)	0.54
Annual Household Income														
<$40,000	REF		REF		REF		REF		REF		REF		REF	
$40,000-$89,999	-0.07 (0.16)	0.67	-0.06 (0.16)	0.69	-0.22 (0.15)	0.16	-0.02 (0.18)	0.89	0.12 (0.17)	0.46	-0.03 (0.14)	0.82	-0.03 (0.17)	0.86
≥$90,000	0.18 (0.18)	0.31	0.34 (0.17)	**0.04**	-0.16 (0.17)	0.32	0.06 (0.19)	0.78	0.43 (0.18)	**0.02**	0.33 (0.15)	**0.03**	0.08 (0.18)	0.65
Comorbid Conditions														
0	REF		REF		REF		REF		REF		REF		REF	
1 or more	-0.32 (0.14)	**0.02**	-0.33 (0.13)	**0.01**	-0.19 (0.13)	0.12	-0.62 (0.15)	**<0.001**	-0.34 (0.14)	**0.02**	-0.32 (0.12)	**0.01**	-0.41 (0.14)	**0.004**
Patient Receipt of Chemotherapy														
No	REF		REF		REF		REF		REF		REF		REF	
Yes	0.44 (0.27)	0.11	0.35 (0.27)	0.19	0.53 (0.26)	**0.04**	0.79 (0.31)	**0.01**	0.29 (0.28)	0.29	0.33 (0.24)	0.17	0.53 (0.28)	0.06
Patient Receipt of Radiation Therapy														
No	REF		REF		REF		REF		REF		REF		REF	
Yes	-0.39 (0.17)	**0.02**	-0.29 (0.16)	0.07	-0.18 (0.16)	0.27	-0.31 (0.19)	0.09	-0.28 (0.17)	0.11	-0.23 (0.15)	0.12	-0.15 (0.17)	0.39
Patient-Reported Cancer Site														
Rectal	REF		REF		REF		REF		REF		REF		REF	

© 2023 Ghazal LV et al. *JAMA Network Open*.

Colon	-0.42 (0.22)	**0.05**	-0.45 (0.21)	**0.03**	-0.06 (0.20)	0.78	-0.18 (0.24)	0.46	-0.23 (0.22)	0.29	-0.39 (0.19)	**0.04**	-0.19 (0.22)	0.39
Both/ unknown	-0.45 (0.19)	**0.02**	-0.37 (0.19)	**0.05**	-0.07 (0.18)	0.72	-0.26 (0.22)	0.23	-0.09 (0.19)	0.63	-0.34 (0.17)	**0.04**	-0.11 (0.19)	0.58

Note. Financial burden is measured as a continuous variable. Models also controlled for partner age, gender, race, education, employment, lost income, and lost work; partner helped by patient financially, patient helped by partner financially; and years since patient diagnosis. Only significant associations are shown.

© 2023 Ghazal LV et al. *JAMA Network Open*.
