# Supplementary material for: Financial Toxicity and Its Association With Health-Related Quality of Life Among Partners of Colorectal Cancer Survivors
Source: JAMA Netw Open. 2023 Apr 6;6(4):e235897. doi: 10.1001/jamanetworkopen.2023.5897 (PMC10080378; doi:10.1001/jamanetworkopen.2023.5897)
Supplement: Supplement 2. — Data Sharing Statement [file jamanetwopen-e235897-s002.pdf]

Data Sharing Statement

Ghazal. Financial Toxicity and Its Association With Health-Related Quality of Life Among Partners of Colorectal Cancer Survivors. *JAMA Netw Open*. Published April 06, 2023. doi:10.1001/jamanetworkopen.2023.5897

Data
Data available: Yes
Data types: Deidentified participant data
How to access data: The datasets generated during and/or analyzed during the current study are available from the corresponding author, at cveenstr@med.umich.edu, on reasonable request.
When available: With publication

Supporting Documents
Document types: None

Additional Information
Who can access the data: Upon reasonable request to researchers whose proposed use of the data has been approved.
Types of analyses: For a specified purpose.
Mechanisms of data availability: After approval of a proposal and with a signed data access agreement.
